# Lemierre's Syndrome Caused by *Klebsiella pneumoniae*: Case Report and Review of the Literature

**DOI:** 10.1002/ccr3.72003

**Published:** 2026-02-05

**Authors:** Abril Aguilar Guerrero, Eduardo Porras Rosales

**Affiliations:** ^1^ Department of Internal Medicine Hospital Escuela Manolo Morales Peralta Managua Nicaragua; ^2^ Infectious Diseases Service Hospital Escuela Manolo Morales Peralta Managua Nicaragua

**Keywords:** diabetes mellitus, internal jugular vein thrombosis, *Klebsiella pneumoniae*, Lemierre's syndrome, odontogenic infection, septic pulmonary emboli, septic thrombophlebitis

## Abstract

*Klebsiella pneumoniae* is an uncommon but increasingly recognized cause of Lemierre's syndrome, a condition classically associated with *Fusobacterium necrophorum.* This report describes a 32‐year‐old woman with poorly controlled type 2 diabetes mellitus who presented with a progressive odontogenic infection complicated by internal jugular vein thrombosis and cavitary septic pulmonary emboli. Imaging demonstrated a deep neck abscess with adjacent thrombophlebitis, and cultures from surgical drainage yielded *K. pneumoniae* with a wild‐type susceptibility profile. The patient required invasive mechanical ventilation, broad‐spectrum antibiotics, surgical source control, and therapeutic anticoagulation, resulting in complete clinical recovery. A review of published cases from 1996 to 2025 was performed, highlighting diabetes as the predominant risk factor, odontogenic or oropharyngeal disease as the most common source, and septic pulmonary emboli as a hallmark manifestation. This case represents the second documented instance of *K. pneumoniae* –associated Lemierre's syndrome in Nicaragua and reinforces the need for early imaging, prompt surgical management, and targeted antimicrobial therapy in diabetic patients presenting with deep neck infections.

## Key Clinical Message

*Klebsiella pneumoniae* represents an emerging atypical etiology of Lemierre's syndrome, particularly among patients with diabetes mellitus. This case highlights the critical role of early diagnostic imaging, prompt surgical source control, and pathogen‐directed antimicrobial therapy in severe odontogenic infections complicated by jugular vein thrombosis and septic pulmonary emboli.

## Graphical abstract

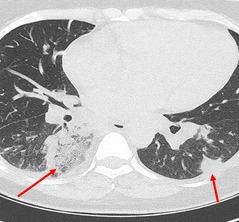

## 1 Introduction

Lemierre's syndrome is an uncommon but potentially life‐threatening condition characterized by septic thrombophlebitis of the internal jugular vein following an oropharyngeal or cervicofacial infection [1, 2]. The classic pathogen, *Fusobacterium necrophorum* , accounts for most cases, particularly in healthy adolescents and young adults [1, 2]. Although its incidence declined with the widespread use of antibiotics, Lemierre's syndrome remains clinically relevant because of its rapid progression, diagnostic challenges, and high risk of severe complications, including cavitary septic pulmonary emboli [1, 2].

In recent years, non‐fusobacterial organisms have emerged as atypical etiologic agents. Among them, *Klebsiella pneumoniae* has become increasingly recognized, especially in patients with poorly controlled diabetes mellitus or other metabolic comorbidities [3, 4, 5, 6, 7, 8, 9, 10, 11, 12, 13, 14, 15]. Hyperglycemia‐induced immune dysfunction may predispose diabetic individuals to aggressive Gram‐negative infections, deep neck suppuration, and extensive thrombosis [3, 4, 5, 6, 7, 8, 9, 10, 11, 12, 13, 14, 15]. Hypervirulent strains (hvKp), particularly K1 serotypes carrying *rmpA*, *rmpA2*, and *magA*, have also been reported, demonstrating the ability to cause invasive, metastatic infections [10]. Compared with classical *Fusobacterium*‐associated disease, *K. pneumoniae* –associated cases often show larger cervical collections, a higher inflammatory burden, and more frequent respiratory compromise, sometimes requiring intensive care support [7, 9, 10, 15]. Early diagnosis therefore depends on maintaining a high index of suspicion, especially in diabetic patients presenting with neck swelling, fever, and respiratory distress. This report describes a severe odontogenic case of *K. pneumoniae* –associated Lemierre's syndrome in a young woman with diabetes mellitus, accompanied by a review of published cases. In this context, *Klebsiella pneumoniae* has been designated by the World Health Organization as a critical priority pathogen in the 2024 Bacterial Priority Pathogens List, emphasizing the urgent global need for improved prevention strategies and effective therapeutic options.

## 2 Case Presentation

A 32‐year‐old woman with a 10‐year history of type 2 diabetes mellitus and arterial hypertension presented with a 15‐day history of right facial pain, dysphagia, intermittent fever, and progressive right‐sided neck swelling. The swelling evolved into a tender, erythematous, warm mass extending from the mandibular border to the thyroid cartilage, accompanied by trismus and increasing discomfort. Three days before admission, she developed worsening dyspnea and pleuritic right‐sided chest pain, prompting emergency evaluation.

On arrival, she was febrile (38.5°C), tachycardic (130 bpm), tachypneic (36 breaths/min), and hypoxemic (oxygen saturation 84% on room air). Her random blood glucose was markedly elevated. Physical examination revealed facial asymmetry, multiple dental foci of infection, and a firm, tender cervical mass involving levels III–IV with overlying erythema and warmth. Respiratory assessment demonstrated the use of accessory muscles and bilateral basal crackles.

On admission, the patient had marked hyperglycemia, with a random blood glucose level of 356 mg/dL and a glycated hemoglobin (HbA1c) of 10.8%, consistent with chronically poor glycemic control. Intensive insulin therapy was initiated during hospitalization, achieving progressive metabolic stabilization without hypoglycemic events.

Arterial blood gas analysis confirmed moderate acute hypoxemic respiratory failure. Her SOFA score was 7 [16] and APACHE II score was 14 [17], indicating significant systemic compromise. Due to progressive respiratory distress, the patient was intubated and placed on invasive mechanical ventilation.

Initial laboratory studies revealed leukocytosis with marked neutrophilia, thrombocytopenia, hyperglycemia, and elevated inflammatory markers (Table 1). Two sets of blood cultures were obtained at admission prior to the initiation of antimicrobial therapy, each consisting of aerobic bottles, in accordance with local laboratory availability. All blood cultures remained negative after standard incubation. Microbiological cultures obtained from surgical drainage of the cervical abscess yielded *Klebsiella pneumoniae*. Antimicrobial susceptibility testing demonstrated a wild‐type susceptibility profile, with sensitivity to third‐generation cephalosporins, carbapenems, β‐lactam/β‐lactamase inhibitor combinations, fluoroquinolones, and aminoglycosides. No extended‐spectrum β‐lactamase production was detected.

**TABLE 1:** Laboratory and arterial blood gas results on admission.

Parameter	Result	Reference range	Units
*Hematology and biochemistry*			
Hemoglobin (Hb)	14.1	12.0–16.0	g/dL
Platelet count	117 × 10^3^	150–400 × 10^3^	/mL
White blood cell count (WBC)	20.9 × 10^3^	4.0–10.0 × 10^3^	/mL
Neutrophils	19.6 × 10^3^	2.0–7.5 × 10^3^	/mL
C‐reactive protein (CRP)	115	< 10	mg/L
Total bilirubin (TB)	1.2	0.2–1.2	mg/dL
Lactate dehydrogenase (LDH)	300	135–225	U/L
Serum creatinine (Cr)	1.3	0.6–1.2	mg/dL
Procalcitonin	11.6	< 0.1	ng/mL
Random blood glucose	356	70–140	mg/dL
HbA1c	10.8	< 6.5	%
*Arterial blood gas (room air)*			
PaO_2_	56	80–100	mmHg
FiO_2_	0.21	—	—
PaCO_2_	34	35–45	mmHg
SaO_2_	84	95–100	%
PaO_2_/FiO_2_ ratio	267	> 300	—

Abbreviations: FiO_2_, fraction of inspired oxygen; HbA1c, glycated hemoglobin; PaCO_2_, partial pressure of carbon dioxide; PaO_2_, partial pressure of oxygen; SaO_2_, oxygen saturation.

**FIGURE 1:**
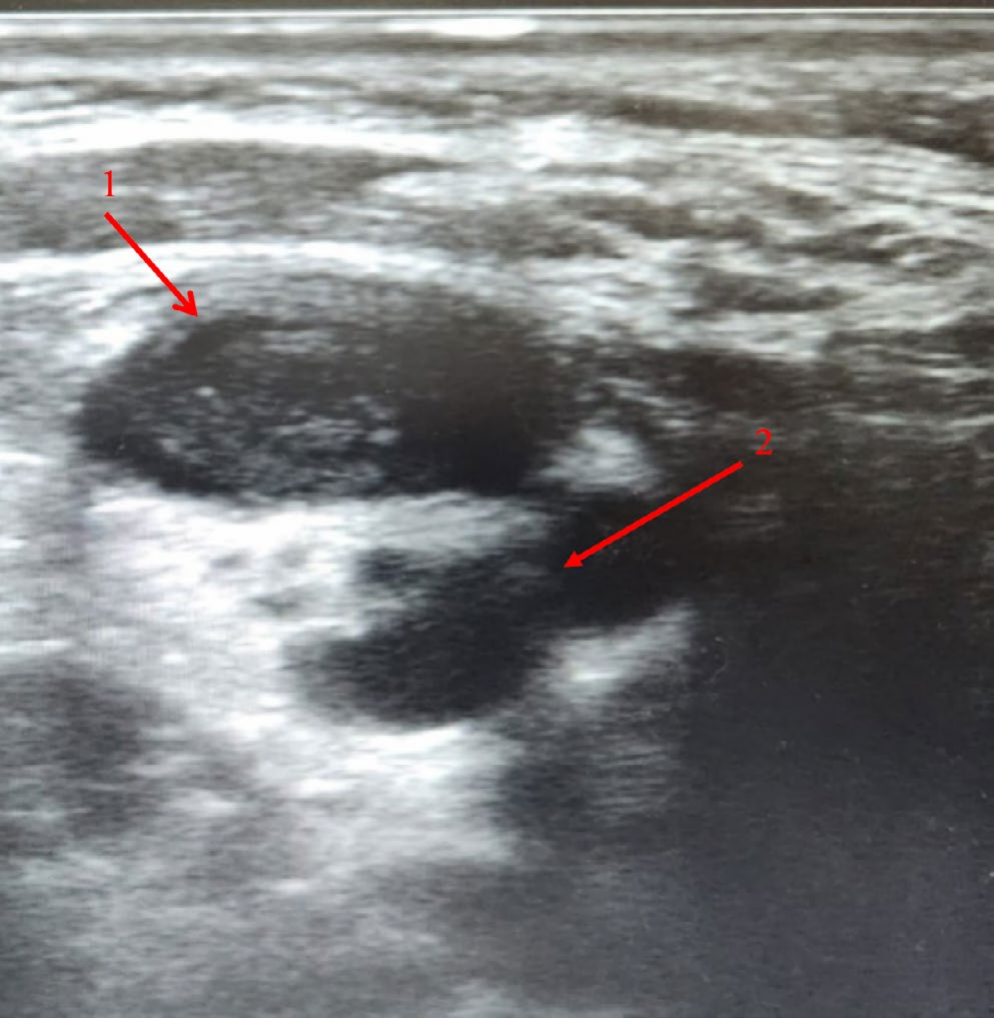
Cervical ultrasonography demonstrating a heterogeneous hypoechoic collection with internal echoes (arrow 1) adjacent to a dilated right internal jugular vein containing heterogeneous echogenic intraluminal material without Doppler flow (arrow 2), consistent with a cervical abscess (approximately 21 mL) and associated internal jugular vein thrombosis.

**FIGURE 2:**
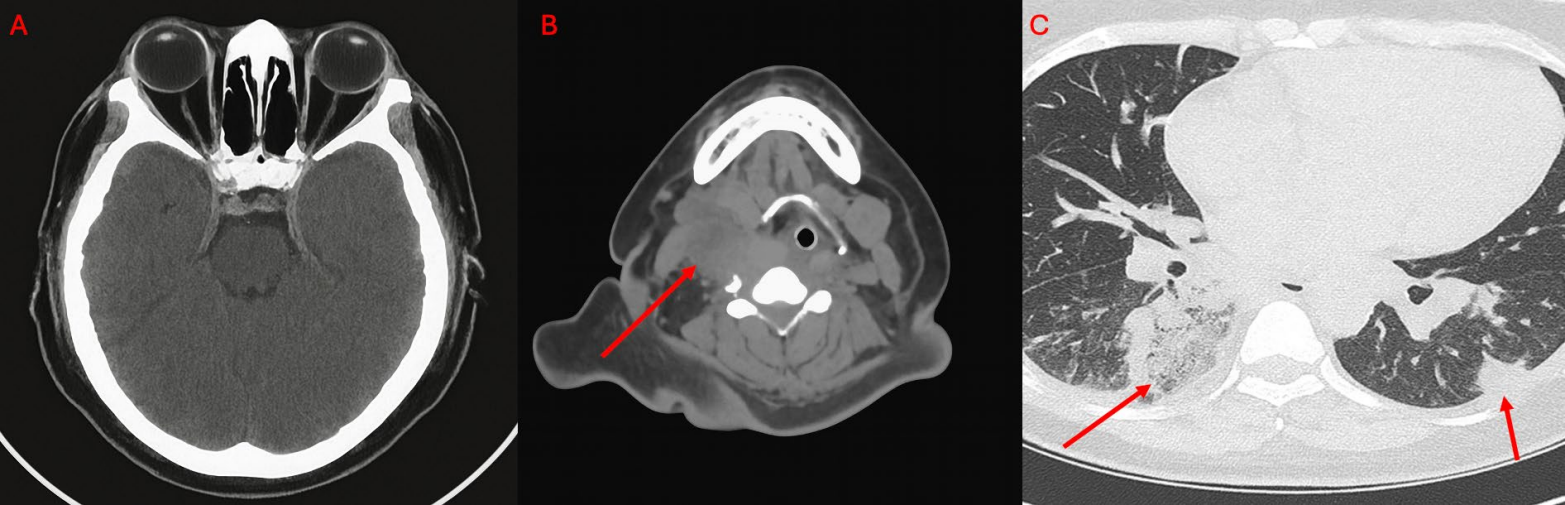
(A) Axial non‐contrast head computed tomography (CT) showing no evidence of intracranial extension, venous sinus thrombosis, or parenchymal abnormalities. (B) Axial contrast‐enhanced neck CT demonstrating a right‐sided hypodense deep neck collection with surrounding soft‐tissue edema and thrombosis of the right internal jugular vein, evidenced by non‐opacified intraluminal material (arrow). (C) Axial contrast‐enhanced chest CT demonstrating multiple peripheral cavitary nodules and consolidations, predominantly in the right lower lobe, consistent with septic pulmonary emboli (arrows).

Cervical ultrasonography demonstrated thrombosis of the right internal jugular vein adjacent to a 21 mL cervical abscess (Figure 1). Chest computed tomography (CT) revealed multiple peripheral cavitary nodules consistent with septic pulmonary emboli, while cranial CT excluded intracranial extension or venous sinus thrombosis (Figure 2).

Because of the evident odontogenic origin, surgical extraction of infected teeth and drainage of the cervical abscess were performed under general anesthesia. Cultures from the drained material grew *Klebsiella pneumoniae* with a wild‐type susceptibility profile, confirming the etiologic agent.

**FIGURE 3:**
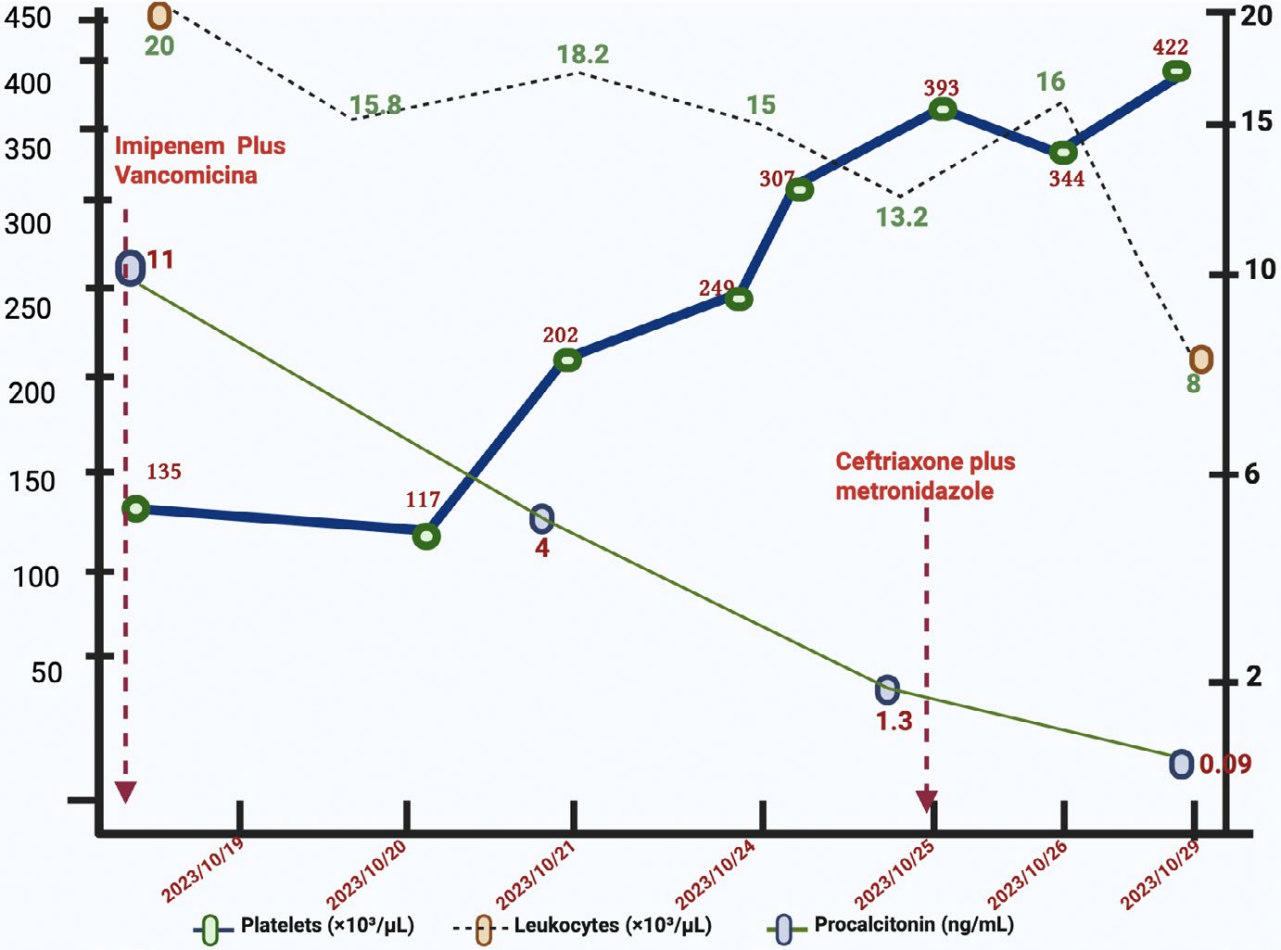
Temporal evolution of leukocyte count, platelet count, and procalcitonin levels during antimicrobial therapy and therapeutic anticoagulation, demonstrating progressive normalization of inflammatory and hematologic parameters. *Figure created with BioRender.com*.

Empiric therapy with imipenem and vancomycin was initiated. Vancomycin was discontinued after microbiological confirmation, and carbapenem therapy was limited to 4 days, followed by targeted treatment with ceftriaxone and metronidazole. Therapeutic anticoagulation with enoxaparin was administered for internal jugular vein thrombosis and was well tolerated, with platelet recovery by day five (Figure 3).

The patient showed steady clinical improvement, became afebrile and normoxemic, and was successfully extubated on day seven. She was discharged on oral amoxicillin–clavulanate with scheduled outpatient follow‐up.

## 3 Investigations and Management

Based on the clinical presentation, targeted diagnostic investigations were promptly initiated, followed by combined medical and surgical management.

The combination of progressive cervical swelling, fever, odynophagia, and respiratory compromise raised several diagnostic possibilities, including deep neck space infection, odontogenic abscess, cervical lymphadenitis, necrotizing fasciitis, Lemierre's syndrome, and septic pulmonary embolism. The presence of a firm, tender cervical mass with overlying inflammatory changes prompted urgent imaging. Cervical ultrasonography revealed thrombosis of the right internal jugular vein adjacent to a deep neck abscess, strongly supporting a diagnosis of Lemierre's syndrome. Chest computed tomography further confirmed multiple cavitated pulmonary nodules consistent with septic emboli, thereby consolidating the diagnosis.

Given the presumed odontogenic origin, empiric broad‐spectrum antimicrobial therapy with imipenem and vancomycin was initiated to ensure coverage of both aerobic and anaerobic organisms. Surgical extraction of the infected teeth and drainage of the cervical abscess provided prompt and effective source control. Culture of the drained material grew *Klebsiella pneumoniae* with a wild‐type susceptibility profile, allowing de‐escalation to targeted therapy with ceftriaxone and metronidazole. Therapeutic anticoagulation with low molecular weight heparin (enoxaparin) was initiated due to extensive internal jugular vein thrombosis and the presence of septic pulmonary emboli. After careful assessment of bleeding risk, anticoagulation was planned for a total duration of 3 months. Follow up cervical ultrasonography demonstrated partial recanalization of the affected vein without hemorrhagic complications.

This combined medical and surgical approach resulted in rapid clinical stabilization, with resolution of fever, improvement in inflammatory markers, and progressive recovery of respiratory function.

## 4 Outcome and Follow‐Up

The patient demonstrated rapid and sustained clinical improvement following cervical drainage, targeted antimicrobial therapy, and therapeutic anticoagulation. Fever resolved within 48 h, and her respiratory status improved progressively, allowing successful extubation on day seven.

At 2 weeks postoperatively, follow‐up cervical ultrasonography revealed partial recanalization of the right internal jugular vein and near‐complete resolution of the cervical abscess. A one‐month follow‐up chest CT showed complete resolution of cavitary pulmonary lesions with no new embolic phenomena. She completed a 14‐day course of intravenous antibiotics followed by an additional 7‐day oral regimen. At her six‐week outpatient evaluation, she remained asymptomatic, with normalized inflammatory markers and no evidence of recurrent infection, thrombosis, or respiratory impairment. She had fully returned to her baseline functional status, and her overall prognosis was favorable.

## 5 Discussion

Lemierre's syndrome is traditionally attributed to *Fusobacterium necrophorum* and most commonly affects previously healthy adolescents and young adults [1, 2]. In recent years, however, *Klebsiella pneumoniae* has emerged as an uncommon yet clinically significant alternative pathogen, predominantly in individuals with metabolic derangements or impaired immune function. Since its initial description in 1996 [3], only 13 cases of *K. pneumoniae* –associated Lemierre's syndrome have been reported worldwide, underscoring both the rarity of this variant and the importance of maintaining diagnostic vigilance.

The epidemiologic profile of this entity differs notably from that of classical Fusobacterium infection. Among the published cases, over 80% of patients had diabetes mellitus, often poorly controlled, highlighting hyperglycemia as a key facilitator of severe Gram‐negative infection [3, 4, 5, 6, 7, 8, 9, 10, 11, 12, 13, 14, 15]. Chronic hyperglycemia compromises fundamental functions of the innate immune response, including neutrophil chemotaxis, oxidative burst, and phagocytosis, thereby predisposing patients to aggressive soft tissue infections and vascular complications. Additional comorbidities reported include alcohol use disorder [10], chronic kidney disease [12], and obesity with diabetic ketoacidosis [14], all of which may further impair host defenses. Collectively, these data point to a distinct phenotype characterized by immunometabolic susceptibility, which appears central to the pathogenesis of *K. pneumoniae* –associated Lemierre's syndrome.

From a microbiological standpoint, most reported isolates demonstrate wild‐type antimicrobial susceptibility. Nevertheless, cases involving hypervirulent *K. pneumoniae* (hvKp) strains have been documented, particularly those harboring the K1 capsular serotype and virulence determinants such as *rmpA*, *rmpA2*, and *magA* [10]. These genetic features confer a hypermucoviscous phenotype that enhances tissue invasion, intravascular persistence, and metastatic dissemination. Importantly, virulence, hypervirulence, and antimicrobial resistance should not be viewed as mutually exclusive phenotypes, as emerging data indicate that certain strains may exhibit overlapping characteristics. Despite global concern regarding antimicrobial resistance in *K. pneumoniae* , only one reported case of Lemierre's syndrome involved an extended‐spectrum β‐lactamase–producing strain [15]. Overall, most infections appear to be community‐acquired and remain susceptible to third‐generation cephalosporins and carbapenems.

Clinically, *K. pneumoniae* ‐associated Lemierre's syndrome mirrors those of the classical form and typically include: fever and systemic inflammatory response, odontogenic or oropharyngeal infection, internal (or, rarely, external) jugular vein thrombosis, and cavitating septic pulmonary emboli, the hallmark metastatic feature [1, 2, 3, 4, 5, 6, 7, 8, 9, 10, 11, 12, 13, 14, 15]. Notably, several patients developed early acute hypoxemic respiratory failure requiring mechanical ventilation [7, 10, 15], a trend that may reflect the heightened inflammatory response characteristic of Gram‐negative sepsis. Severity scoring systems such as SOFA [16] and APACHE II [17] often reveal early organ dysfunction at presentation. Contrast‐enhanced CT imaging remains the gold standard for identifying jugular vein thrombosis and pulmonary embolic lesions, whereas ultrasonography provides a valuable rapid assessment tool, particularly in unstable patients.

Analysis of the 13 previously reported cases (Table 2) reveals a consistent clinical and epidemiologic pattern: Diabetes mellitus is the predominant risk factor, odontogenic and oropharyngeal infections are the most frequent sources [3, 4, 6, 13]. Internal jugular vein thrombosis is the defining vascular complication, although external jugular involvement has been documented [14]. Cavitating septic pulmonary emboli are common and serve as the characteristic metastatic manifestation [3, 4, 5, 6, 7, 8, 9, 10, 11, 12, 13, 14, 15].

**TABLE 2:** Reported cases of *Klebsiella pneumoniae* ‐associated Lemierre's syndrome (1996–2025).

Author/year	Country	Patient age/sex	Risk factors	Source of infection	Jugular vein	ABX susc	Treatment	SPE	Outcome	References
Bhagat et al. 1996	USA	52, M	DM	Odontogenic	IJV	WT	Cefazolin → Cefuroxime (14 days)	Yes	Survived	[3]
Wu et al. 2012	Taiwan	54, F	DM	Oropharyngeal	IJV	WT	Ceftriaxone + Metronidazole (21 days)	Yes	Survived	[4]
Garbati et al. 2012	Saudi Arabia	63, M	DM	Deep neck abscess/oropharyngeal	IJV	WT	Clindamycin + cefuroxime + warfarin (6 weeks)	No	Survived	[5]
Chuncharunee et al. 2015	Thailand	51, F	DM	Right parapharyngeal abscess	IJV	WT	Ceftazidime → ampicillin/sulbactam; later meropenem; + multiple surgical drainages; + enoxaparin	Yes	Died (septic shock + multidrug‐resistant *A. baumannii* )	[6]
Chen et al. 2023	Taiwan	43, M	DM, Alcoholic liver cirrhosis	Right neck soft tissue infection (no clear pharyngeal focus stated)	IJV	WT	Meropenem	Yes	Died (respiratory failure within 24 h of ICU transfer)	[7]
Sabaka et al. 2019	Slovakia	19, M	Previously healthy	Tonsillopharyngitis → cervical abscess	Left IJV thrombosis	WT	Ceftriaxone + Clindamycin → Piperacillin–tazobactam + Clindamycin (21 days) + surgical drainage → oral Moxifloxacin (7 days)	No	Survived; complete recovery	[8]
Hwang et al. 2021	South Korea	56, F	Uncontrolled DM (HbA1c 11%), HTN, chronic otitis media	Oropharyngeal infection (sore throat) → parotid region nodule	No IJV thrombosis	WT	Piperacillin–tazobactam + Levofloxacin → Meropenem + Vancomycin → Meropenem + Amikacin; anticoagulation (LMWH → Apixaban); antifungals (Amphotericin → Itraconazole)	Yes	Survived (prolonged hospitalization; rehospitalized but recovered)	[9]
Lee SE et al. 2021	USA	63, F	HTN, hyperlipidemia, well‐controlled DM	Right oropharyngeal/lateral pharyngeal infection; reactive lymphadenopathy	Right IJV extension into retromandibular vein	WT, hvKp‐K1	Piperacillin–tazobactam → Meropenem → Ampicillin–sulbactam IV → Amoxicillin–clavulanate PO (6 weeks) + Rivaroxaban (3 months)	Yes	Survived	[10]
Ngo et al. 2024	Vietnam	62, M	DM	Left neck abscess (multiloculated); oropharyngeal mass effect	Left IJV	No ABX susc.	Meropenem + Vancomycin; surgical drainage; glycemic control; vasopressors; mechanical ventilation	Yes	Survived; extubated day 4; discharged day 15	[11]
Rangan et al. 2024	India	48, M	DM	Odontogenic focus	Rigth IJV	WT	Piperacillin–tazobactam + Metronidazole + Amikacin; surgical I and D; LMWH anticoagulation; glycemic control	No	Survived	[12]
Wong et al. 2025	Malaysia	54, F	DM	Odontogenic infection → deep neck abscesses (parapharyngeal & submandibular) + mandibular osteomyelitis	Right IJV	WT	IV amoxicillin–clavulanate (2 weeks) → PO amoxicillin–clavulanate (6 weeks total); surgical drainage; LMWH → Rivaroxaban anticoagulation	No	Survived	[13]
Treminio & De la Cruz 2025	Nicaragua	65, F	DM, Hypertension, obesity, DKA on admission	Left submandibular abscess + otitis with otorrhea + pharyngotonsillar exudate	Left EJV	WT	Imipenem–cilastatin + Vancomycin → Ciprofloxacin (per susceptibilities) + Enoxaparin → Warfarin + surgical drainage.	Yes	Survived	[14]
Lee WS et al. 2012	Taiwan	56, F	Previously healthy	Left neck abscess	Left IJV	ESBL	Ceftazidime + Metronidazole → Ceftriaxone + Amikacin → Meropenem (monotherapy) → Meropenem + Fosfomycin (IV) for 2 months	Yes^a^	Survived	[15]
Present case, 2025	Nicaragua	32, F	DM	Odontogenic	Right IJV	WT	Imipenem/Vancomycin → Ceftriaxone + Metronidazole → Oral amoxicillin–clavulanate (21 days)	Yes	Survived	—

Abbreviations: CKD, chronic kidney disease; DM, diabetes mellitus, EJV, external jugular vein; F, Female; hvKp, hypervirulent *Klebsiella pneumoniae* ; IJV, internal jugular vein; K1, capsular serotype; M, male; MDR, multidrug resistant; Metro, metronidazole; Piperacillin–Tazo, piperacillin–tazobactam; PO, oral administration; SPE, septic pulmonary emboli.

^a^ Multiple diffuse brain abscesses + seizures.

Management strategies emphasize early antimicrobial therapy and definitive source control. Empirical antimicrobial regimens should provide coverage against aerobic Gram‐negative organisms and anaerobes. Most reported patients received carbapenems, third‐generation cephalosporins, or β‐lactam/β‐lactamase inhibitor combinations, with subsequent de‐escalation guided by susceptibility results proving consistently effective [3, 4, 5, 6, 7, 8, 9, 10, 11, 12, 13, 14, 15]. Treatment duration varied according to disease severity, ranging from 14 to 21 days in uncomplicated cases [3, 4, 8] to 3–6 weeks in patients with extensive deep neck infection or respiratory compromise, and up to 6–8 weeks in the single ESBL associated case complicated by intracranial dissemination [15]. These observations underscore the need for individualized therapy guided by clinical response and radiologic follow‐up.

Odontogenic and oropharyngeal infections represent the most frequent primary foci, underscoring the importance of rapid and definitive surgical source control [3, 4, 6, 13]. Prompt extraction of infected teeth and drainage of deep neck abscesses are essential to reduce bacterial burden, resolve thrombophlebitis, and prevent ongoing embolization. In the present case, timely surgical intervention played a pivotal role in achieving clinical stabilization.

The role of anticoagulation in Lemierre's syndrome remains controversial, as robust randomized controlled data are lacking. However, large observational studies and systematic analyses suggest that selected patients with extensive internal jugular vein thrombosis, thrombus propagation, or recurrent septic embolization may benefit from anticoagulation, without a clear increase in major bleeding events [18, 19]. Careful individualized assessment of bleeding risk is therefore essential. In our patient, anticoagulation was initiated due to extensive jugular thrombosis and septic pulmonary emboli, maintained for 3 months, and guided by follow‐up imaging demonstrating partial recanalization without adverse events.

*Klebsiella pneumoniae* ‐associated Lemierre's syndrome often follows a more severe clinical course than classical Fusobacterium infection. Several cases have reported early respiratory failure requiring mechanical ventilation [7, 10, 15], and two fatalities (≈15%) have been documented [6, 7, 20]. Poor glycemic control, delayed diagnosis, and extensive cavitating septic pulmonary emboli appear to be key predictors of adverse outcomes.

This report represents the second documented case of *K. pneumoniae* ‐associated Lemierre's syndrome in Nicaragua, following the case involving external jugular thrombosis described by Treminio and De la Cruz [14]. Together, these cases suggest that Gram‐negative variants of Lemierre's syndrome may be underrecognized in Central America, highlighting the need for early imaging, aggressive source control, and targeted antimicrobial therapy, particularly among diabetic patients.

A structured literature search conducted across PubMed/MEDLINE, EMBASE, Scopus, Web of Science, and Google Scholar from January 1990 to January 2025 identified 13 published cases of *K. pneumoniae* associated with Lemierre's syndrome, which contextualize the present report within the existing body of evidence.

Overall, this case underscores *Klebsiella pneumoniae* as an emerging atypical cause of Lemierre's syndrome, particularly in the setting of poorly controlled diabetes mellitus. Compared with classical *Fusobacterium necrophorum‐associated* disease, Gram‐negative variants may present with more severe local infection, extensive thrombosis, and early respiratory failure. Early recognition, prompt imaging, definitive surgical source control, appropriate antimicrobial therapy, and selected use of anticoagulation were essential for a favorable outcome. Increased awareness of non‐fusobacterial Lemierre's syndrome is crucial to avoid diagnostic delay and improve prognosis in high‐risk patients.

## Author Contributions

**Eduardo Porras Rosales:** conceptualization, data curation, formal analysis, funding acquisition, investigation, methodology, supervision, writing – review and editing. **Abril Aguilar Guerrero:** conceptualization, formal analysis, investigation, methodology, project administration, writing – original draft, writing – review and editing.

## Funding

The authors have nothing to report.

## Consent

Written informed consent was obtained from the patient for the publication of this case report, including all clinical information, diagnostic data, and accompanying imaging studies. The patient explicitly approved the use of de‐identified photographs, radiologic images, and any other potentially identifiable material in accordance with the journal's patient consent requirements and international ethical standards.

## Conflicts of Interest

The authors declare no conflicts of interest.

## Data Availability

No additional datasets were generated or analyzed for this case report. All relevant clinical information is included within the manuscript.

## References

[1] A. Tiwari , “Lemierre's Syndrome in the 21st Century: A Literature Review,” Cureus 15 (2023): e43685, 10.7759/cureus.43685. 37724228 PMC10505273

[2] K. Kuppalli , D. Livorsi , N. J. Talati , and M. Osborn , “Lemierre's Syndrome due to *Fusobacterium necrophorum* ,” Lancet Infectious Diseases 12, no. 10 (2012): 808–815, 10.1016/S1473-3099(12)70089-0. 22633566

[3] B. Bhagat , M. Ahmed , S. W. Ali , and K. Roistacher , “A Case of Lemierre's Syndrome due to *Klebsiella pneumoniae* ,” Infectious Diseases in Clinical Practice 5, no. 6 (1996): 389–390.

[4] H. M. Wu , Y. J. Tsai , J. C. Lin , and P. R. Hsueh , “A Lemierre Syndrome Variant Caused by *Klebsiella pneumoniae* ,” Journal of the Formosan Medical Association 111, no. 7 (2012): 403–405, 10.1016/j.jfma.2012.03.012. 22817819

[5] M. A. Garbati , A. M. Ahsan , and A. M. Hakawi , “Lemierre's Syndrome due to *Klebsiella pneumoniae* in a 63‐Year‐Old Man With Diabetes: A Case Report,” Journal of Medical Case Reports 6 (2012): 97, 10.1186/1752-1947-6-97. 22472458 PMC3337804

[6] A. Chuncharunee and T. Khawcharoenporn , “Lemierre's Syndrome Caused by *Klebsiella pneumoniae* in a Diabetic Patient: A Case Report and Literature Review,” Hawaii Journal of Medicine & Public Health 74, no. 8 (2015): 276–279. PMC4536737 26279962

[7] T. A. Chen , Y. T. Chuang , H. Y. Lin , and C. H. Chen , “Lemierre's Syndrome Caused by *Klebsiella pneumoniae*: A Case Report and Literature Review,” Cureus 15, no. 8 (2023): e44434, 10.7759/cureus.44434. 37664341 PMC10469873

[8] P. Sabaka , M. Káčerik , M. Bendžala , and H. Káčerová , “Lemierre Syndrome Caused by *Klebsiella pneumoniae* Complicated by Epidural Abscess – Case Report,” IDCases 19 (2019): e00664, 10.1016/j.idcr.2019.e00664. 32226757 PMC7093747

[9] S. Y. Hwang , S. J. Shin , and H. E. Yoon , “Lemierre's Syndrome Caused by *Klebsiella pneumoniae* : A Case Report,” World Journal of Nephrology 10, no. 5 (2021): 101–108, 10.5527/wjn.v10.i5.101. 34631480 PMC8477271

[10] S. E. Lee , A. Mushtaq , M. Gitman , et al., “Lemierre's Syndrome Associated With Hypervirulent *Klebsiella pneumoniae*: A Case Report and Genomic Characterization,” IDCases 25 (2021): e01173, 10.1016/j.idcr.2021.e01173. 34141583 PMC8188389

[11] T. D. Ngo , C. T. Nguyen , and N. Ho , “Lemierre's Syndrome due to *Klebsiella pneumoniae* Results in Pulmonary Abscess Complications in a Patient With Diabetes: A Rare Case Report,” Case Reports in Infectious Diseases 2024, no. 1 (2024): 8176530, 10.1155/crdi/8176530. 39741700 PMC11685315

[12] N. M. Rangan , A. K. Singh , R. C. Yadav , et al., “Lemierre Syndrome due to *Klebsiella pneumoniae*: A Rare Case Report With Review of Literature,” Journal of Rare Diseases 3 (2024): 30, 10.1007/s44162-024-00054-x.

[13] J. W. Wong , J. Y. Pwi , S. P. Lau , and C. Y. Chang , “Presentation of Lemierre's Syndrome Secondary to *Klebsiella pneumoniae*‐Caused Neck Abscess Following an Odontogenic Infection,” Cureus 17, no. 7 (2025): e87882, 10.7759/cureus.87882. 40809662 PMC12344742

[14] Y. J. Treminio Juárez and N. la De Cruz Vega Jarquin , “Atypical Lemierre Syndrome With External Jugular Thrombophlebitis: A Case Report,” IDCases 42 (2025): e02428, 10.1016/j.idcr.2025.e02428. 41323571 PMC12664341

[15] W. S. Lee , F. D. Wang , Y. H. Shieh , S. O. Teng , and T. Y. Ou , “Lemierre Syndrome Complicating Multiple Brain Abscesses Caused by Extended‐Spectrum β‐Lactamase‐Producing *Klebsiella pneumoniae* ,” Journal of Microbiology, Immunology, and Infection 45, no. 1 (2012): 72–74, 10.1016/j.jmii.2011.09.012. 22154996

[16] J. L. Vincent , A. de Mendonça , F. Cantraine , et al., “Use of the SOFA Score to Assess the Incidence of Organ Dysfunction/Failure in Intensive Care Units: Results of a Multicenter, Prospective Study,” Critical Care Medicine 26, no. 11 (1998): 1793–1800, 10.1097/00003246-199811000-00016. 9824069

[17] W. A. Knaus , E. A. Draper , D. P. Wagner , and J. E. Zimmerman , “APACHE II: A Severity of Disease Classification System,” Critical Care Medicine 13, no. 10 (1985): 818–829, 10.1097/00003246-198510000-00009. 3928249

[18] M. López , T. Chen , P. Ramírez , et al., “Patients With Lemierre's Syndrome Have a High Risk of New Thromboembolic Complications, Clinical Sequelae, and Death: An Analysis of 712 Cases,” Journal of Internal Medicine 289, no. 3 (2020): 332–343, 10.1111/joim.13111. 32445216

[19] J. Ge , P. Zhou , Y. Yang , et al., “Anticoagulation May Contribute to Antimicrobial Treatment of Lemierre's Syndrome: A Case Report,” Thrombosis Journal 19 (2021): 6, 10.1186/s12959-021-00336-0. 34736465 PMC8567340

[20] R. Kreuzpointner , L. Valerio , G. Corsi , et al., “Ophthalmic Complications of Lemierre's Syndrome,” Acta Ophthalmologica 100, no. 5 (2021): 510–516, 10.1111/aos.14871. 33829646

